# Autologous cell vaccine as a post operative adjuvant treatment for high-risk melanoma patients (AJCC stages III and IV)

**DOI:** 10.1038/sj.bjc.6600251

**Published:** 2002-05-03

**Authors:** M Lotem, T Peretz, O Drize, Z Gimmon, D Ad EL, R Weitzen, H Goldberg, I Ben David, D Prus, T Hamburger, E Shiloni

**Affiliations:** Sharett Institute of Oncology, Hadassah University Hospital, Jerusalem, Israel 91120; Department of Surgery, Hadassah University Hospital, Jerusalem, Israel 91120; Department of Plastic Surgery, Hadassah University Hospital, Jerusalem, Israel 91120; Department of Oncology, Sheba Medical Center, Ramat Gan, Israel 52662; Department of Oncology, Rambam Medical Center Haifa, Israel 31096; Department of Pathology, Hadassah University Hospital, Jerusalem, Israel 91120; Department of Surgery B, Carmel Medical Center, Haifa, Israel 34362

**Keywords:** autologous melanoma vaccine, adjuvant treatment, delayed type hypersensitivity

## Abstract

This study evaluates the overall survival and disease free survival of melanoma patients that were treated with an autologous melanoma cell vaccine, administered as a post-operative adjuvant. Included are 43 patients with totally resected metastatic melanoma (28-AJCC stage III, 15-AJCC stage IV), with a median follow up of 34 months (6–62). The treatment consisted of eight doses of a vaccine made of 10–25×10^6^ autologous melanoma cells either released from the surgical specimen or grown in cell cultures. Tumour cells were conjugated with hapten dinitrophenyl, mixed with Bacille Calmette Guérin and irradiated to 110 Gy. Both disease free survival and overall survival were found to be correlated with intensity of evolving delayed type hypersensitivity to subcutaneous injection of unmodified melanoma cells. Patients with a delayed type hypersensitivity reaction of ⩾10 mm had a median disease free survival of 17 months (mean 35 months) and a mean overall survival of 63 months (median not reached). In contrast, patients with a negative or weak delayed type hypersensitivity had a median disease free survival of 9 months (relative risk of recurrence=4.5, *P*=0.001), and a median overall survival of 16 months (relative risk of death=15, *P*=0.001). Stage III patients with a positive delayed type hypersensitivity reaction had an improved disease free survival of 16 months and a mean overall survival of 38 months, whereas patients with a negative delayed type hypersensitivity had a median disease free survival of 7 months (relative risk=4.5, *P*=0.02) and a median overall survival of 16 months (relative risk=9.5, *P*=0.005). The adjuvant administration of autologous melanoma vaccine was associated with improved disease-free and overall survival to selected patients who successfully attained anti-melanoma reactivity as detected by positive delayed type hypersensitivity reactions to unmodified melanoma cells.

*British Journal of Cancer* (2002) **86**, 1534–1539. DOI: 10.1038/sj/bjc/6600251 www.bjcancer.com

© 2002 Cancer Research UK

## 

Patients suffering from malignant melanoma with metastases to regional lymph nodes, satellites and metastases in-transit are defined as stage III (The new American Joint Committee on Cancer (AJCC) classification) (Balch *et al*, 2000). More than half of these patients die of their disease within 2 years (Cascinelli *et al*, 1984), and the prospects are even worse for patients with distant metastases (AJCC stage IV). Even when surgical excision is feasible, the 2 year survival rates for these patients range only from 14 to 26% (Eton *et al*, 1998). Presently, the issue of adjuvant treatment for these high-risk patients is unsettled. Based on ECOG and intergroup studies E1684 (Kirkwood *et al*, 1996), E1690 (Kirkwood *et al*, 2000) and E1694 (Kirkwood *et al*, 2001), the authors stress that accumulated data reveal reduction in the hazard ratio for relapse and death for patients (AJCC stage IIB and III) treated with maximally tolerated dose of interferon-alfa 2b. Opponents, however, doubt whether the considerable toxicity of this protocol justifies the therapeutic advantage (Spitler, 2001). Furthermore, premature analysis of EORTC No. 18952 study hints towards emerging survival benefit for patients treated with an intermediate-low dose of IFN-alfa 2b whereas the intermediate-high dose schedule is not superior to control (Eggermont, 2001). Overall, the adjuvant potential of immunotherapeutic strategies is supported by these studies.

Melanoma vaccines are an attractive alternative approach for an adjuvant treatment. It is true that their value in metastatic disease is limited: the response rates range only from 5 to 15%, and these positive responses are mainly limited to subcutaneous metastases (Mitchell *et al*, 1990; Morton *et al*, 1992; Sato, 1996; Eton *et al*, 1998). In spite of this, a much larger percentage of patients treated with melanoma vaccines develop definite immune reactions. Although in most cases these reactions fail to translate into clinical tumour regressions, improved survival has been repeatedly reported for those patients who develop anti-melanoma immune responses (Bystryn *et al*, 1992; Livingston *et al*, 1994; Hsueh *et al*, 1998; Baars *et al*, 2000). These observations have led to several ongoing studies for the evaluation of the adjuvant role of active immunotherapy for high-risk melanoma patients. The assumption is that the early induction of anti-melanoma immunity might prolong disease free and/or overall survival of AJCC stage III and IV patients suffering from malignant melanoma.

One approach of active immunotherapy has been developed by Berd and colleagues. Their vaccine is composed of autologous melanoma cells conjugated with a hapten-dinitrophenyl (DNP), irradiated and mixed with Bacille Calmete Guérin (BCG). They administered this vaccine as an adjuvant treatment to patients with AJCC stage III and stage IV resectable nodal disease. In comparison to control patients, this method has yielded improved disease free and overall survival (Berd *et al*, 1997).

Encouraged by earlier work of this group, we chose to use this method of Berd and colleagues and to further extend it by using melanoma cell cultures from the primary tumour. This modification of the method allowed us to include patients with small tumour volumes into the program. Here we report our results with the first 43 stage III and IV cutaneous and non-cutaneous melanoma patients whom we treated using this approach as a postoperative adjuvant therapy for resectable disease. We did not include a no-treatment arm in our study and therefore will be unable to calculate the risk reduction ratio of the group. Nevertheless, a strong prognostic marker became apparent during the follow up period: patients who developed positive skin reaction to subcutaneous injection of their own melanoma cells faired significantly better than those who failed to do so. We suggest that their improved survival attests to the adjuvant effect of autologous melanoma vaccine.

## PATIENTS AND METHODS

### Patients

The patients included in this study were grouped in two categories: (1) Twenty-eight patients with the modified AJCC stage III cutaneous malignant melanoma metastatic to the regional lymph node (macrometastases, N1-3/b), five of them also having satellites and metastases in transit (stage IIIC); (2) 15 patients with cutaneous malignant melanoma metastatic to viscera (AJCC stage IV) or with metastatic non-cutaneous melanoma (2-rectal melanoma, 4-ocular melanoma, 1-laryngeal melanoma). For clinical details see Table 1

**Table 1:**
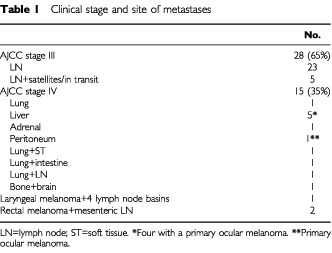
Clinical stage and site of metastases

.

To be eligible for our study, patients had to have undergone complete surgical excision of metastatic disease and be found disease free based on whole body CT scan performed less than 3 weeks prior to surgery. In addition, patients had to have a positive skin reaction to at least one out of four recall antigens: PPD, candidin, trychophytin, and tetanus toxoid. A positive reaction was defined as an erythema larger than 5 mm in diameter. The study was approved by the Institutional Review Board and informed consent was obtained before any therapy was administered.

Four of the patients received radiotherapy after surgery and prior to the initiation of the vaccinations. One of these received whole brain irradiation for a single brain metastasis, and the other three received radiation treatment to the abdominal wall or to cervical lymph nodes for close margins and for extracapsular lymph node invasion. We started the vaccine procedure for these four patients 1 month after their radiotherapy was completed. Although these patients received radiotherapy prior to their inclusion in our study, they remained immune competent, which we ascertained by their skin tests. After the resection of their liver metastases, two patients with ocular melanoma had received four courses of an intra-arterial hepatic infusion of fotemustine before we started the vaccine procedure. Four patients were referred to us with metastatic disease after they failed to respond to either low dose (one patient) or high dose (three patients) interferon-α adjuvant therapy.

### Vaccine preparation

Tumour specimens were procured fresh and sterile. For large tumour bulks, cells were extracted by mechanically or by enzymatic dissociation with collagenase and DNAse (Sigma, St. Louis, MO, USA), frozen in a controlled rate freezer, and stored in liquid nitrogen in a medium containing 2.5% human albumin and 20% DMSO until needed. If the total cell yield was less than 200×10^6^ cells, cell suspensions were put into culture bottles with Dulbecco's Modified Eagle's Medium (DMEM, Gibco BRL, Gaithersburg, MD, USA), 10% foetal calf serum (Gibco BRL), HEPES (1 : 500), pen-strep (1 : 100) and glutamin (1 : 100) and expanded to the required number, necessary for preparation of at least eight vaccine doses, of 10–25×10^6^ cells each. Culturing the cells resulted in the preferential selection of melanoma cells, so that the vaccine finally consisted of purified melanoma cells.

When we were unable to dissociate the cells mechanically – as in the case of a small specimen or a dense connective tissue – tumours were cut into pieces of less than 1 mm^3^ in size, put on a 60 mm^2^ plastic dish (Nunc), and then glued to the dish with drops of foetal calf serum (Gibco BRL). Culture medium was added to the dish 24 h later. When cell growth was observed at the periphery of tumour pieces, we transferred the cell mass to a culture bottle. To assure melanocytic progeny, staining of slides covered by cultured cells was performed with monoclonal antibodies S-100 and HMB-45, using the alkaline phosphatase anti-alkaline phosphatase (APAAP) reaction (Zymed Inc., San Francisco, CA, USA). Positive staining of more than 50% of cells with at least one of these antibodies was required.

On treatment day, the cells were thawed, washed and irradiated to 110 Gy. A sample was stained with Trypan blue and counted after irradiation. Conjugation of melanoma cells with DNP was performed by the method of Miller and Claman (1976). Briefly, melanoma cells were washed with Hanks balanced solution-no HSA, resuspended to a concentration of 5×10^6^ ml^−1^, mixed, incubated for 30 min and washed again. Prior to vaccination an appropriate amount of BCG was added, starting with a dilution of 1 : 50 and reaching 1 : 500–1000, according to the resulting granuloma at vaccination site.

### Vaccination procedure (see Table 2)

**Table 2:**
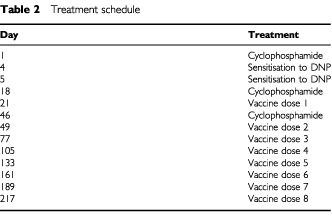
Treatment schedule

On day 1, patients received 300 mg m^2^ of intravenous cyclophosphamide. On days 4 and 5, patients were sensitised to DNP by applying 0.1 ml of 2% DNP dissolved in acetone-corn oil (Sigma) topically to the inner aspect of the arm. On day 18, patients received a second dose of 300 mg m^2^ cyclophosphamide. On day 21, the prepared vaccine was injected into three adjacent sites on the upper arm or thigh, avoiding limbs where lymph node dissection had been previously performed. Seven additional doses of the vaccine were administered at intervals of 21–28 days. Before injecting the second dose of the vaccine, a third dose of 300 mg m^3^ of cyclophosphamide was administered i.v. Patients were evaluated periodically every 2 months. These evaluations included a physical examination, a complete blood count, and liver function tests. A chest X-ray was performed every 3 months and a total body CT scan was made every 6 months.

### DTH evaluation

Skin testing to evaluate delayed type hypersensitivity to autologous melanoma cells was performed by intradermal injection of 1–3×10^6^ unmodified melanoma cells irradiated to a dose of 170 Gy. To avoid contamination with foreign proteins and chemicals, the cells were triple washed before injection. DTH was measured by the maximal diameter of erythema at 48 h after the injection of the vaccine. Erythema smaller than 5 mm was defined as a negative reaction. We arbitrarily chose the value of 10 mm of erythema to discriminate between weak DTH (<10 mm) and strong DTH (⩾10 mm). Patients were also skin tested to 3×10^6^ unmodified autologous lymphocytes.

### Statistical analysis

The analysis focused on the impact of DTH on OS and DFS. The OS and DFS were estimated by the Kaplan-Meier's method. Significance was calculated by the log rank test. To further validate the effect of DTH on survival, a Cox proportional hazards method was used to adjust for the following covariates: age (⩽60, >60 years), gender, time elapsing from surgery to the first vaccination (⩽3, >3 months), and stage (III, IV). A relative risk (RR) of more than 1 denotes the increased risk of death or recurrence for patients with a negative or a weak DTH response as compared to patients with a strong positive DTH, calculated by the Cox model. The model was built for all patients (adjusted for stage) and for stage III patients only, because of the importance of this group.

## RESULTS

All of the patients that we entered into the study have been included in our analysis. Thirty-three (73%) of these patients completed full schedules; 11 (27%) of the patients suffered disease recurrence while on treatment. Median follow up period is 34 months (range: 6–62)

### General results

Using the Kaplan-Meier method and the log rank test, the single parameter that was strongly correlated in a univariate analysis with overall survival (OS) and disease-free survival (DFS) was the DTH response. The Cox proportional hazards model, adjusted for age, gender, time from surgery and stage confirmed this finding (Table 3

**Table 3:**
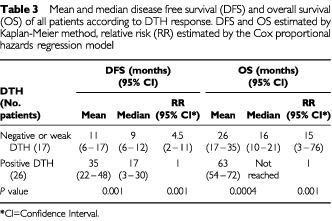
Mean and median disease free survival (DFS) and overall survival (OS) of all patients according to DTH response. DFS and OS estimated by Kaplan-Meier method, relative risk (RR) estimated by the Cox proportional hazards regression model

). DFS and OS were analysed for DTH <10 mm (negative or weak DTH) *vs* DTH ⩾10 mm (strong positive DTH). Twenty-six patients (60%) attained strong positive DTH whereas 17 patients (40%) had only weak or negative DTH. Patients who failed to attain a strong DTH had an increased risk of 15 (95% confidence interval (CI) 3–76; *P*=0.001) to die of their disease and a relative risk of 4.5 (95% CI 1.8–11; *P*=0.001) to have a disease recurrence. Current patient status with respect to DTH response is shown in Figure 1

**Figure 1:**
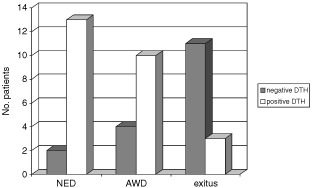
Current patient status with respect to DTH response. The number of patients in each of three categories. NED: no evidence of disease; AWD: alive with disease; Exitus: deceased. In each of these categories, the patients were divided into two groups according to the pattern of the delayed type hyper-sensitivity (DTH).

**Figure 2:**
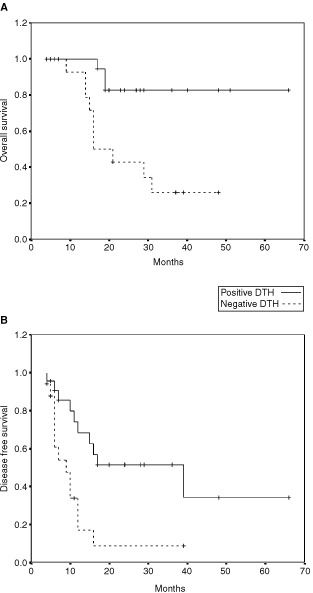
Kaplan-Meier estimates of (**A**) overall survival and (**B**) disease free survival as a function of DTH status for all patients.

. For the whole group, the mean DFS was 24 months (median 12 months) and the mean OS was 48 months (restricted for 62 months). Fortunately, at this time the median OS has not been reached. Patients that attained a DTH reaction ⩾10 mm a median DFS of 17 months, and a mean OS of 63 months (median not reached yet). Patients that failed to attain DTH or had only a weak reaction had a median DFS of 9 months and a median OS of 16 months (Table 3, Figure 2

).

Interactions between DTH and the covariates of age, gender, time from surgery and stage for OS and DFS were tested and found not significant using the Cox model with an interaction term.

### Stage III patients

The vaccine was administered to 28 AJCC stage III patients whose median DFS was 12 months and median OS was 31 months. Patients that attained a DTH reaction >10 mm had a median DFS of 16 months and a mean OS of 38 months (restricted for 48 months, median not reached). Patients with a negative or weak (<10 mm) DTH had a median DFS of 7 months and a median OS of 16 months (Table 4

**Table 4:**
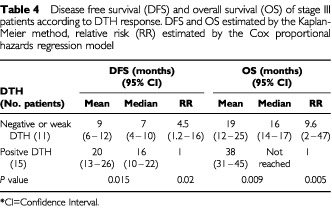
Disease free survival (DFS) and overall survival (OS) of stage III patients according to DTH response. DFS and OS estimated by the Kaplan-Meier method, relative risk (RR) estimated by the Cox proportional hazards regression model

). Using the Cox model, the estimated RR of death for patients who failed to attain positive DTH was 9.6 (95% CI 2–47; *P*=0.005) and the RR for recurrence was 4.5 (95% CI 1.2–16; *P*=0.02).

### Stage IV patients

The vaccine was administered to 15 AJCC stage IV cutaneous and non-cutaneous, metastatic melanoma patients, whose median DFS was 12 months and median OS was not reached yet (mean 56 months). This group is too small and heterogenous to be sub analysed. However, even for this small group, differences in OS and DFS according to patients' DTH status almost reach statistical significance. All eight patients with positive DTH are alive.

One patient with ocular melanoma metastatic to the liver, who had intra-hepatic artery infusions of fotemustine, had extremely strong DTH reactions (erythema and oedema of >30 mm in diameter); this patient has been disease-free for 47 months. Another patient, with rectal melanoma and metastatic mesenteric lymph nodes, had a DTH reaction of 45 mm. She has been disease free for 54 months; her last DTH testing, done 36 months after the completion of full vaccination schedule, still revealed a 35 mm erythema.

### Toxicity

No significant systemic toxicity was observed in any of the patients. Vaccination sites resolved leaving depressed atrophic scars.

## DISCUSSION

Stage III and IV melanoma patients face a grave prognosis. The overall 2-year survival for patients with lymph node metastases (stage III) is 29–38% and their median DFS is 12 months. Patients with distant metastases do even more poorly. Eton (1998a) reported that the 2-year survival for patients with one organ involvement is 14%, for multiple subcutaneous metastases – 17%, and for lung metastases alone – 26%. The median survival period for these stage IV patients was reported to be 7.5, 15 and 8 months, respectively (Barth *et al*, 1995). Most patients who can be rendered apparently ‘disease free’ by surgical excision of all detectable disease, harbour micrometastases and eventually develop evident metastases and succumb to their disease.

Attempts towards improving the dismal prognosis of these patients by adjuvant conventional therapies, that is chemotherapy with DTIC, have not proven to be beneficial. This discouraging situation has stimulated researchers to seek different approaches, of which the use of immunotherapy is one of the most compelling. There are many examples supporting the suggestion that the immune system has an important role in the development of anti-melanoma reactivity. These examples include cases of spontaneous regression of primary and metastatic melanoma, and also the development of vitiligo in melanoma patients. These phenomena have been accompanied by various circulating anti-melanocyte antibodies and melanoma specific cytotoxicity conveyed by tumour infiltrating lymphocytes (Naughton *et al*, 1983; Bottger *et al*, 1992; Kawakami *et al*, 1994). Immunotherapy is most likely to eradicate metastatic disease when the tumour burden is minimal and when the host has not yet become immunosuppressed either by tumour itself (Akiyama *et al*, 1983), or by various chemotherapeutic agents.

Various vaccination strategies against melanoma are based on presenting melanoma-associated antigens (MAAs) or peptides to elicit specific cytotoxicity against tumour cells (Hersey *et al*, 1986; Bystryn *et al*, 1992; Livingston *et al*, 1994; Mitchell *et al*, 1997; Eton *et al*, 1998b; Hsueh *et al*, 1998; Rosenberg *et al*, 1998). The source of these antigens can either be whole or fragmented melanoma cells or, alternately, purified peptides, specific of melanoma.

Theoretically, the advantage of using autologous melanoma cell vaccines is that the diverse antigens in the vaccine would be presented in the context of the patient's own Major Histocompatibilty Complex (MHC). This condition is mandatory for the recognition of cytotoxic T lymphocytes (CTLs) (Imro *et al*, 1999). To date, the largest published study on the use of autologous melanoma vaccine as a post-operative adjuvant for stage III cancer patients is the study by Berd and colleagues that they undertook in 1997 (Berd *et al*, 1997). They reported survival rates for vaccinated patients that were significantly higher than those of control patients treated by surgery alone. Vaccinated patients that became DTH positive had significantly improved survival rates (71% 5-year survival) compared to patients that remained DTH negative (49% 5-year survival). Overall, the projected 5-year DFS rates for all their (DTH positive and DTH negative) vaccinated patients was 45% and the projected 5-year OS rate was 58%. The toxicity associated with these treatments was negligible. Latest results of an expanded population of 214 patients and extended follow-up show a 5 year survival rate of 47%. Similar to our group, Berd *et al* (2001) has repeated the observation that the induction of DTH to unmodified tumour cells was associated with significantly longer 5-year survival (DTH ⩾5 mm=69%, DTH <5 mm=33%, *P*<0.001). A disadvantage of using autologous melanoma vaccines is that it is not always feasible to use the autologous tumour to prepare the vaccine: the tumour specimens may be small, containing only a small number of intact tumour cells. In an attempt to solve this problem, we cultured cells from the original tumours. Using these cell cultures allowed us to enlarge the number of cells available for vaccination. Moreover, by using cultured cells we could minimise or even avoid using tissue degrading enzymes, which might lead to changes in cell surface molecules. By passaging the cultured cells two to five times we assured that the vaccine would include only viable melanoma cells, and that any remaining undesirable enzymes from the dissociation procedure would be diluted and removed. Furthermore, culturing the tumour cells resulted in the preferred reproduction of melanoma cells, thus assuring that necrotic cells and associated non-tumour cells would be excluded from the vaccine.

Though our study did not include a control group, the results disclose a significant and relevant correlation between the development of a DTH response to unmodified, autologous melanoma cells and disease free survival (DFS). A median DFS of 17 months for patients with a strong positive DTH reaction is significantly better than the median DFS of 9 months for the non-reactive patients (Table 3). In stage III patients, the respective mean OS was 19 *vs* 38 months and the mean DFS – 9.6 *vs* 20 months. These differencies were found to be statistically significant.

Though we did not originally design our study to discern survival benefit for patients, over a median follow up period of 34 months we attained an overall cancer-related survival ratio of 76% (33/43, with two cancer-unrelated deaths), considerably higher than reported. It is important to note that patients eligible for adjuvant treatment with an autologous vaccine are in an unfavourable sub-group for their stage of disease. For a surgeon to recommend such treatment, stage III patients must have a macroscopic disease (Nb).

The correlation between DTH and survival has been observed by the research groups of Berd *et al* (1986, 1991, 1993) and Baars *et al* (2000) using autologous melanoma vaccines, and by the groups of Morton (1992) using allogeneic melanoma vaccines and of Bystryn (1992), using shed material melanoma vaccines. DTH is considered to be a classical CD4+ T cell response, but both CD4+ and CD8+ T cell subsets with anti melanoma reactivity were isolated from skin biopsies of DTH sites (Waanders *et al*, 1997). Conversion from a non-reactive status to a reactive one is associated with the emergence of MAA-reactive CTLs. We do not consider the DTH to be a mere representation of an intact cell mediated immunity of the individual, because before being vaccinated each one of our patients was tested with four common antigens and demonstrated at least one 5 mm wide reaction. Injections of autologous lymphocytes cryopreserved with 10% foetal calf serum caused no skin reaction in any of our patients, suggesting that their reactions were melanoma specific. The BCG that was given as an immunoadjuvant, has probably no independent significant therapeutic effect (Tan *et al*, 1993).

In conclusion, we found that the adjuvant administration of autologous melanoma vaccine was associated with the improved disease-free and overall survival of selected patients that successfully attained anti-melanoma reactivity as detected by positive DTH reactions to injected unmodified melanoma cells. Melanoma cell cultures permit this method to be used for patients with small tumour specimens and yield a purified tumour cell population. Obviously, our present study was carried out without any control groups. With this compromise, the study still supports the idea that an adjuvant vaccination strategy holds promise for the treatment of high risk melanoma patients.
